# Unpassivated edges as gateways to collision-driven topological transformations in finite graphene nanoflakes

**DOI:** 10.1140/epjd/s10053-026-01242-8

**Published:** 2026-09-01

**Authors:** Klaudia Cielinska, Alexey V. Verkhovtsev, Cauê P. Souza, Matthew D. Dickers, Andrey V. Solov’yov, Nigel J. Mason, Felipe Fantuzzi

**Affiliations:** 1 School of Engineering, Mathematics and Physics, University of Kent, Park Wood Rd, Canterbury, CT2 7NH UK; 2 MBN Research Center, Altenhöferallee 3, 60438 Frankfurt am Main, Germany; 3 School of Natural Sciences, University of Kent, Park Wood Rd, Canterbury, CT2 7NH UK

## Abstract

**Abstract:**

Finite graphene nanoflakes occupy a unique position in the carbon allotrope landscape. Near equilibrium, these systems exhibit pronounced structural robustness, indicating that large-scale allotropic transformations require strongly non-equilibrium energy deposition. In this work, we investigate how unpassivated graphene edges act as chemically active gateways for collision-driven topological transformations in finite graphene systems. Using reactive molecular dynamics simulations, we model energetic collisions involving a C$_{60}$ fullerene projectile and a finite C$_{296}$ graphene nanoflake across a broad parameter space of collision energies, incidence angles, and edge orientations. The structural response is analysed through statistical transformation maps that quantify the probability of nanoflake bending, edge stitching, and the formation of nanotube-like structures. We identify a regime of collision energies and oblique incidence angles under which localised energy deposition at unpassivated edges promotes bond rearrangement, cooperative bending, and covalent stitching between opposing edges. Furthermore, we find that armchair-directed impacts align opposing zigzag edges and promote efficient edge-to-edge stitching over a broader parameter range. In contrast, zigzag-directed impacts are associated with a higher probability for atom ejection and bring opposing armchair edges into contact, which is less favourable for seam propagation, resulting in a narrower parameter window for nanotube-like structure formation. These results establish a non-equilibrium framework linking local edge chemistry and collision-induced energy deposition to topological evolution in finite graphene systems. The results demonstrate collision-driven self-organisation as a viable pathway for the emergence of tubular nanostructures without reliance on catalytic surfaces or sustained thermal annealing.

**Graphic abstract:**

**Figure:**
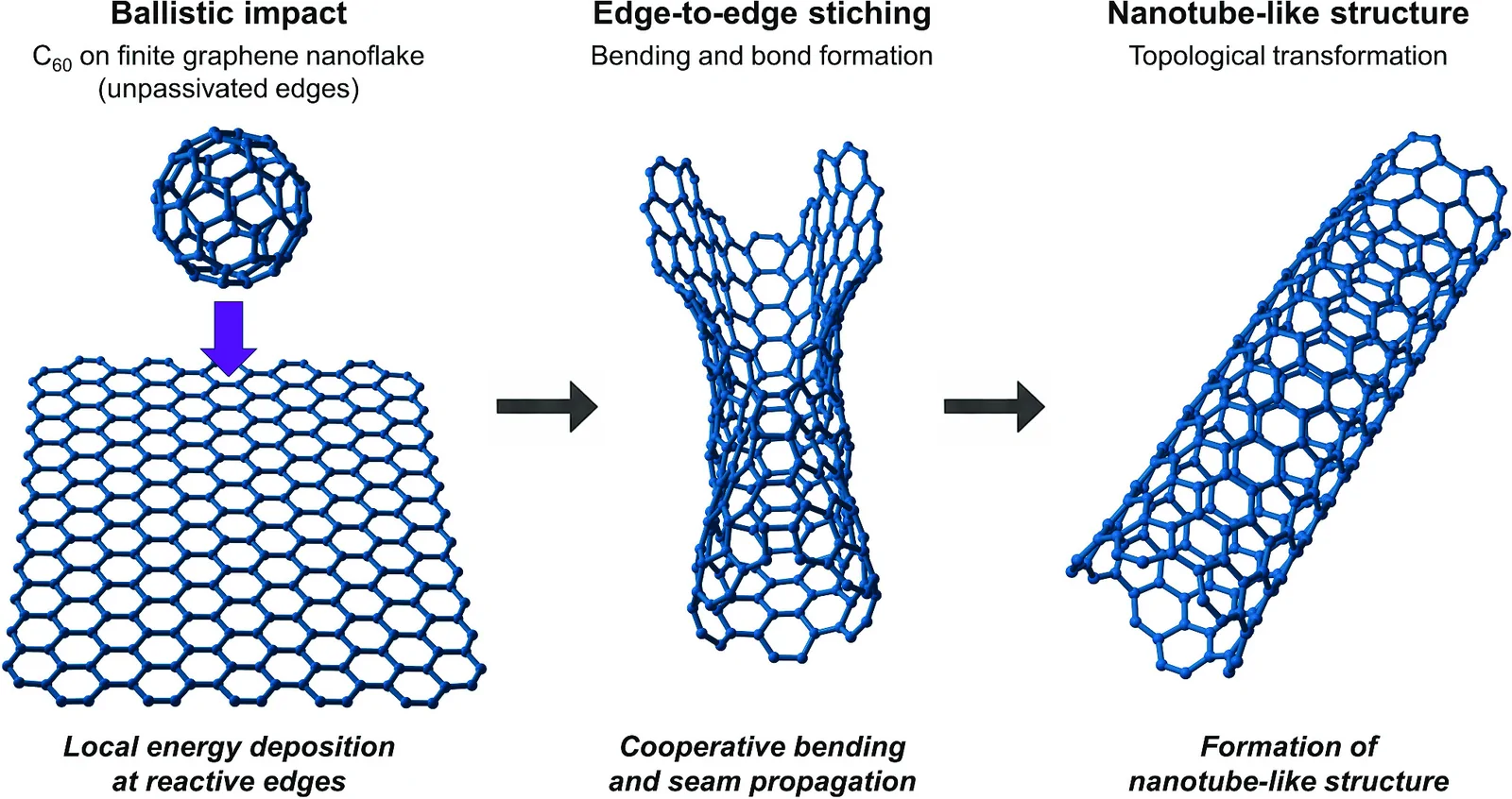

## Introduction

Carbon is an exceptionally versatile element, capable of forming a broad spectrum of allotropes whose structural, electronic, and mechanical properties span a wide range. These include three-dimensional networks such as diamond [1], layered and tubular structures such as graphite [2] and carbon nanotubes [3], as well as zero- and two-dimensional nanostructures including fullerenes [4, 5] and graphene [6]. This diversity originates from the ability of carbon to adopt multiple hybridisation states (*sp*, $sp^2$, and $sp^3$) and to form stable covalent bonds across a wide range of coordination environments. Among these allotropes, graphene—a two-dimensional sheet of $sp^2$-bonded carbon atoms arranged in a honeycomb lattice—has emerged as a paradigmatic low-dimensional material. Its massless Dirac-like charge carriers [7, 8], exceptionally high carrier mobility [6, 9–11], large thermal conductivity [12–14], mechanical robustness [15, 16], and maximal surface-to-volume ratio [17] have made it a central platform for the development of next-generation electronic [18], optoelectronic [19–21], spintronic [22, 23], and photocatalytic [24, 25] technologies.

Beyond canonical device-oriented applications, graphene and its derivatives have also been explored as multifunctional material platforms, including reinforcement phases in graphene–polymer composites [26–28], active components in photocatalytic systems [25] and in the photocatalytic degradation of environmental pollutants [29], and as nanocarriers for drug delivery and biomedical applications [30]. Graphene-based materials have further enabled highly sensitive detection schemes for neglected tropical diseases, including biosensors for dengue [31] and Zika [32] viruses, while graphene oxide has been shown to inhibit malaria parasite invasion at the cellular level [33]. From a fundamental perspective, graphene has also become a model system for exploring electron confinement [34], topological defects [35], and the coupling between lattice structure and electronic response [36]. While much of the early research focused on extended graphene sheets, increasing attention has shifted towards finite graphene fragments, commonly referred to as graphene nanoflakes (GNs) [37] or graphene quantum dots [38]. In these systems, spatial confinement and the presence of edges introduce quantum and topological degrees of freedom that are absent in bulk graphene: finite-size effects open electronic band gaps, while geometric shape and edge topology influence the spatial distribution of frontier orbitals, localised electronic states, and magnetic response [39–42].

As a consequence, the properties of GNs reflect not only the characteristics of the basal plane but also the interplay between size, shape, topology, and the chemical activity of their edges. In this broader context, graphene quantum dots have emerged as a distinct class of materials, exhibiting size- and shape-dependent optical, electronic, and optoelectronic properties that can be tuned through synthesis parameters and elemental doping [43]. Finite graphene-based nanostructures have also found applications in biological and biomedical contexts, including interactions with *Trypanosoma brucei*, the causative agent of human African trypanosomiasis [44, 45], commonly referred to as sleeping sickness, where graphene quantum dots have been investigated as platforms for the targeted modulation of parasite mitochondrial function [46].

From a structural and materials perspective, GNs occupy a unique position in the carbon allotrope landscape as intermediates between planar graphene, closed fullerene cages, and tubular carbon nanotubes. This dual role as both quantum-confined systems and structural building blocks has motivated long-standing hypotheses that finite graphene fragments may serve as precursors for other carbon nanostructures [47], provided that suitable non-equilibrium mechanisms exist to induce curvature, bond rearrangement, and topological closure. Computational and experimental studies have proposed multiple transformation routes linking finite graphene fragments, nanotube segments, and closed cages: large-radius carbon nanotube fragments can unfold into metastable graphene-like patches, while the inverse process can lead to the closure of finite fragments into fullerene cages through the progressive incorporation of non-hexagonal rings and local bending [48–50]. Complementary studies have further shown that polycyclic aromatic hydrocarbon and nanoflake intermediates can act as building blocks in the assembly of fullerenes and soot, with their geometry and flexibility influencing whether closure into cages or aggregation into extended networks is favoured [51–53].

Transformations between planar, tubular, and closed carbon nanostructures involve bond rearrangements such as Stone–Wales-type rotations and the incorporation of non-hexagonal rings, which induce bending and enable topological closure [54, 55]. These processes are typically associated with substantial activation barriers and strong coupling between mechanical strain and electronic structure [56]. Despite these theoretical pathways, both experimental and computational studies have demonstrated that GNs remain remarkably stable under near-equilibrium conditions, preserving their planar geometry through edge reconstructions and vibrational relaxation rather than transforming into closed or tubular forms [57]. This thermal robustness indicates that near-equilibrium pathways alone are insufficient to drive large-scale allotropic transformations in finite graphene systems.

Strongly non-equilibrium conditions therefore emerge as viable drivers of structural transformations in GNs. High-energy impacts at the nanoscale can generate intense, localised stimuli, including transient pressure spikes and rapid bond deformation, accompanied by ultrafast energy redistribution throughout the structure [58–60]. These effects activate bond-reorganisation pathways that remain inaccessible under steady-state conditions, making ballistic collisions a powerful means of probing the energy landscape connecting different carbon allotropes [61]. Such mechanisms are also relevant in astrochemical and planetary environments, where carbonaceous species are exposed to cosmic rays, energetic ions, and grain–grain collisions at temperatures that can fall well below $10~\textrm{K}$ [62].

A central factor in these transformations is the chemical nature of graphene edges. In finite nanoflakes, undercoordinated edge atoms possess reactive dangling bonds that serve as nucleation sites for bond reconstruction, structural bending, and closure. Experimental and theoretical studies have shown that hydrogen-free edges exhibit enhanced reactivity, whereas hydrogen passivation suppresses the driving force for tubular formation [47, 63, 64]. In this context, the present study investigates collision-induced allotropic transformations in finite, hydrogen-free GNs using classical reactive molecular dynamics simulations. We model high-energy impacts between a C$_{60}$ fullerene projectile and a finite C$_{296}$ nanoflake, systematically varying the impact energy and incidence angle to quantify edge-mediated effects, transformation statistics, and structural closure pathways [65].

Although this computational framework could be extended to passivated nanoflakes, hydrogen termination is expected to inhibit direct stitching by saturating the dangling bonds required for inter-edge bond formation. Closure would therefore likely require collision-induced removal or rearrangement of the terminating groups to expose reactive carbon sites before seam propagation can occur. By linking impact conditions, edge reactivity, and structural response, this work establishes an atomistic framework for understanding how finite graphene systems with unpassivated edges traverse the energy landscape connecting planar, tubular, and closed carbon allotropes, with implications for nanoscale materials processing and the non-equilibrium chemistry of carbon in astrophysical environments.

## Computational details

**Fig. 1:**
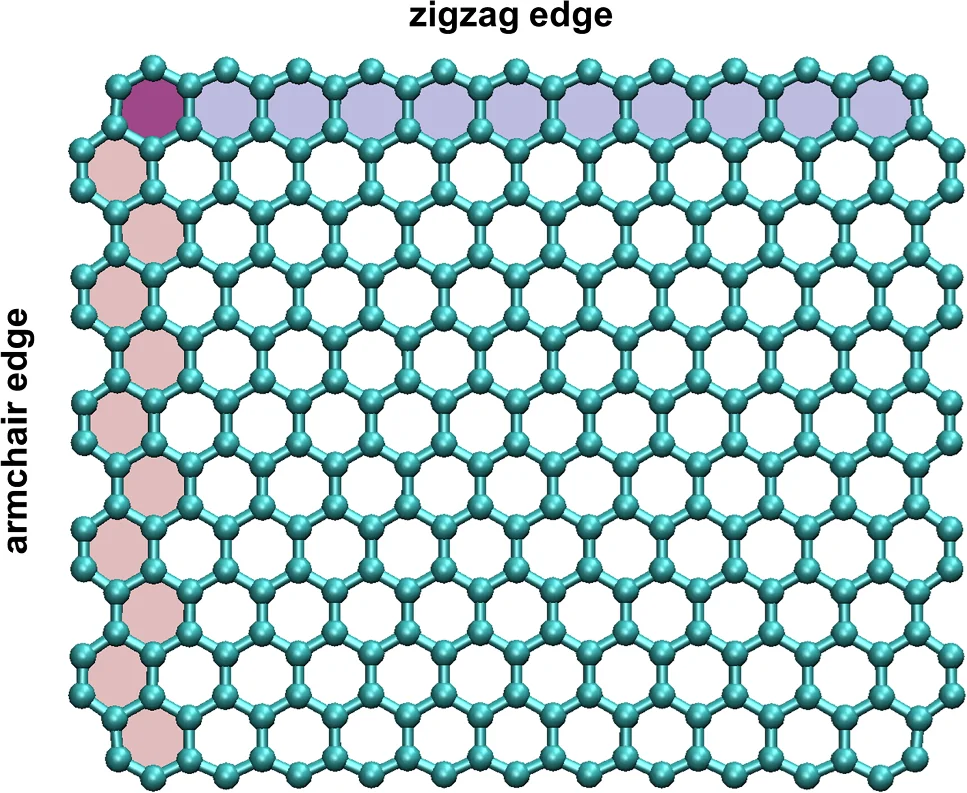
Relaxed initial geometry of the square C$_{296}$ graphene nanoflake used as the collision target. Armchair and zigzag edge segments (11 of each) are highlighted, showing the balanced edge composition and planar structure prior to impact

All simulations were performed using MBN Explorer [66], a software package for multiscale simulations of the structure and dynamics of complex meso-bio-nano (MBN) systems, including irradiation [67] and collision-driven [61] processes. The MBN Studio toolkit [68] was used to create the initial system geometries and prepare the necessary input files. The simulated system consisted of a finite GN containing 296 carbon atoms (C$_{296}$) and a spherical fullerene projectile (C$_{60}$). The nanoflake was constructed as a planar square fragment with well-defined and evenly distributed armchair and zigzag edges (11 segments of each type), which were left unpassivated to retain chemically reactive dangling bonds and structural flexibility under impact (Fig. 1).

The nanoflake dimensions (23.865 Å along the zigzag edge and 29.376 Å along the armchair edge) were selected to achieve an approximate target-to-projectile mass ratio of five, ensuring efficient momentum transfer and substantial local energy deposition at the impact site while maintaining sufficient lateral extent to support cooperative bending, long-range strain propagation, and edge-to-edge interaction across the nanoflake. At the same time, this size remains computationally feasible while enabling statistically reliable ensemble sampling. The aspect ratio of the resulting nanotube-like structures is inherently constrained by the dimensions of the initial nanoflake. In principle, the same collision-driven bending and edge-stitching mechanism could operate for elongated graphene nanoflakes, with the longer dimension determining the nanotube length and the transverse dimension largely determining its diameter. However, increasing the flake length would require deformation and seam formation to propagate coherently over a greater distance, potentially altering the favourable collision conditions and the timescale required for complete closure.

Nanoflakes of different shapes, including triangular and hexagonal ones, were also examined in preliminary simulations. However, the nearly square C$_{296}$ nanoflake was selected for the systematic analysis because it contains equal numbers of armchair and zigzag edge segments, enabling a balanced statistical comparison between the two collision orientations. Nanoflake size and shape are expected to influence the bending response, edge-to-edge separation, and propagation of the stitching seam. The quantitative energy and angular windows reported here are therefore specific to the present target, and a systematic investigation of these finite-size and shape effects remains an important direction for future work.

Initial nanoflake coordinates were adapted from a curated nanostructure dataset [69] and optimised using the velocity quenching algorithm prior to impact to eliminate residual strain and ensure structural stability. All numerical tolerances were set to $1\times 10^{-5}$ and were fully satisfied upon convergence. The C$_{60}$ projectile was positioned at an initial separation of 40 Å from the nominal impact point, measured from its centroid. This impact point was placed at a distance equal to half the fullerene radius from the selected nanoflake edge in order to favour edge-mediated bending and bond formation rather than central perforation of the flake. An additional vertical offset of 2 Å above the nanoflake surface was introduced to account for the finite interaction range of the force field and to suppress van der Waals pre-contact prior to the onset of the ballistic phase. Here, the term *ballistic* denotes inertia-driven motion in which the projectile propagates freely under microcanonical (NVE) conditions without the influence of external fields or dissipative media and does not imply penetration or permanent embedding into the target. A schematic representation of the nanoflake geometry and edge definitions is shown in Fig. 1. To account for variations in the local atomic alignment and initial collision geometry, the impact point was randomly displaced along the direction of the projectile velocity vector projected onto the nanoflake plane within $\pm ~0.71~{{\AA }}$ (approximately half a C–C bond length in the flake), and the projectile was randomly rotated prior to each simulation. Collisions directed towards armchair- and zigzag-oriented edges were treated as separate ensembles.

Short-range C–C interactions within the system were described using the many-body Brenner potential [70], which provides the energetic description of covalent bond breaking, bond formation, and changes in carbon coordination. Long-range non-bonded interactions between the fullerene projectile and the graphene nanoflake were represented using a Lennard–Jones potential with the parameters reported in Ref. [71]. Changes in molecular topology during the simulations were monitored and quantified using the reactive CHARMM (rCHARMM) approach [72]. Following Ref. [61], this auxiliary interaction was assigned a negligibly small bond dissociation energy of $10^{-4}~\mathrm {kcal\,mol^{-1}}$ and a bond-length threshold of $2.0~{{\AA }}$. It therefore contributes negligibly to the forces and does not compete with the Brenner potential but enables the dynamic identification of bond formation, bond rupture, non-hexagonal rings, and edge stitching. The present setup is conceptually related to the adaptive intermolecular reactive empirical bond-order (AIREBO) potential [73], which combines a REBO bond-order term with non-bonded and torsional interactions within a unified framework. Here, these functions are treated separately: the Brenner and Lennard–Jones potentials describe the energetics, whereas the negligibly small rCHARMM bonded term provides the connectivity information required for topological analysis.

Both the nanoflake and the projectile were initialised at $0~\textrm{K}$, and no thermostat was applied. This protocol isolates the mechanical response to ballistic energy deposition and preserves a direct link between localised kinetic-energy transfer, structural bending, and topological rearrangement. At finite temperature, background lattice fluctuations, edge reconstruction, thermally activated wrinkling, and defect annealing could modify the kinetics of edge contact and seam propagation, potentially shifting the quantitative collision-energy and incidence-angle windows for closure. The present results therefore describe the limiting case of initially cold nanoflakes.

For a prescribed collision energy *E*, the projectile velocity was determined from $v=\sqrt{2E/m}$, where *m* is the mass of the C$_{60}$ projectile, equal to 60 times the mass of a carbon atom, and was decomposed according to the selected incidence angle. MD simulations were performed for $15~\textrm{ps}$, which was sufficient to capture the ballistic phase, rapid non-equilibrium atomic dynamics, and subsequent relaxation into metastable or transformed morphologies. Selected cases were extended up to $200~\textrm{ps}$ to verify the post-formation structural stability of nanotube-like configurations.

Collision energies were sampled over 100–$1000~\textrm{eV}$ in increments of $50~\textrm{eV}$, and incidence angles were varied between 10$^\circ$ and 60$^\circ$, measured with respect to the nanoflake plane, on a non-uniform grid. For each combination of edge type, energy, and angle, ten statistically independent trajectories were generated, yielding a total of 5700 collision events.

Structural classification was performed by aligning the final configurations to reference nanotube geometries using rigid-body transformations (translations and rotations only). The reference structures were constructed by topologically closing the same C$_{296}$ nanoflake and relaxing the resulting geometries with the same interaction model. Owing to the finite dimensions of the parent nanoflake, these structures are not commensurate with ideal nanotubes of unique (*n*, *m*) chirality, but their diameters (approximately 8.2 Å and 9.8 Å, respectively) and local bonding patterns are closest to those of finite armchair-like (6,6) and zigzag-like (12,0) nanotubes, respectively, and are therefore used as geometric nanotube-like templates.

Atoms ejected from the nanoflake as a result of the fullerene impact were removed prior to analysis, and the root-mean-square deviation (RMSD) was used as a quantitative similarity metric. Transformations into nanotube-like structures were identified using an RMSD threshold of 2 Å relative to the corresponding reference nanotube geometry. This value was selected by comparing the RMSD with visual and topological inspection of representative final structures. Because the threshold is substantially smaller than the reference nanotube diameters of approximately 8.2 and 9.8 Å, it accommodates the local distortions expected in short, finite nanotubes while generally excluding partially closed, open, strongly defective, or globally non-tubular configurations. Structures with RMSD values close to the threshold were regarded as borderline cases and interpreted together with their visual and topological characteristics.

A separate geometric attachment criterion, based on the separation of the centres of mass of the projectile and nanoflake, was used to identify persistent composite structures. Each trajectory was initially classified into one of four mutually exclusive outcome categories: (i) attached configurations, in which the fullerene remains bound to the nanoflake; (ii) nanotube-like structures satisfying the RMSD criterion; (iii) intact non-tubular structures without atom ejection; and (iv) fragmented structures characterised by atom ejection. Across the entire range of collision energies and incidence angles explored in this work, no persistent attachment (i.e. adsorption of the fullerene to the nanoflake) was observed. Consequently, the attachment channel is neglected in the statistical analysis that follows.

For each combination of collision energy *E*, incidence angle θ, and nanoflake edge orientation α, the resolved probability of nanotube-like formation was computed as

1 $$\begin{aligned} P_{\textrm{CNT}}(E,\theta ,\alpha ) = \frac{N_{\textrm{CNT}}(E,\theta ,\alpha )}{N_{\textrm{total}}(E,\theta ,\alpha )}, \end{aligned}$$

where $N_{\textrm{CNT}}(E,\theta ,\alpha )$ denotes the number of trajectories terminating in nanotube-like structures and $N_{\textrm{total}}(E,\theta ,\alpha )$ is the total number of trajectories for the specified conditions. The probabilities of atom-ejection events, $P_{\textrm{ej}}(E,\theta ,\alpha )$, and intact non-tubular outcomes, $P_{\textrm{int}}(E,\theta ,\alpha )$, are defined analogously. By construction, for each set of collision parameters $(E,\theta ,\alpha )$, these probabilities form a complete partition of the observed outcomes, satisfying

2 $$\begin{aligned} P_{\textrm{CNT}}(E,\theta ,\alpha ) + P_{\textrm{ej}}(E,\theta ,\alpha ) + P_{\textrm{int}}(E,\theta ,\alpha ) = 1. \end{aligned}$$

Angle-resolved probabilities, $P(\theta ,\alpha )$, were obtained by averaging $P(E,\theta ,\alpha )$ over all sampled collision energies, while energy-resolved probabilities, P(E,α), were obtained by averaging over all sampled incidence angles. These reduced representations provide angle-resolved and energy-resolved views of the transformation patterns, whereas the fully resolved probabilities $P(E,\theta ,\alpha )$ retain the complete dependence on collision energy, incidence angle, and edge orientation.

Since the outcome probabilities sum to unity within each $(E,\theta ,\alpha )$ ensemble, the probability $P_{\textrm{CNT}}$ increases at the expense of competing channels, primarily atom ejection and intact non-tubular relaxation pathways, providing a direct measure of how different collision conditions lead to distinct structural transformations of the system.

## Results and discussion

This section is organised as follows. In Sect. 3.1, we establish a topological classification of collision outcomes, identifying the dominant classes of metastable morphologies and their dependence on edge orientation, incidence angle, and collision energy. In Sect. 3.2, we map the probability of successful transformation to nanotube-like structures across the full parameter space, delineating the angular and energetic windows that favour cooperative bending and continued edge-to-edge stitching. Finally, in Sect. 3.3, we resolve the underlying sequence of events through time-resolved trajectory analysis, revealing how localised edge reactivity, elastic deformation of the finite flake, and post-closure energy redistribution combine to accomplish coherent topological closure.

### Collision outcome landscape and topological diversity

**Fig. 2:**
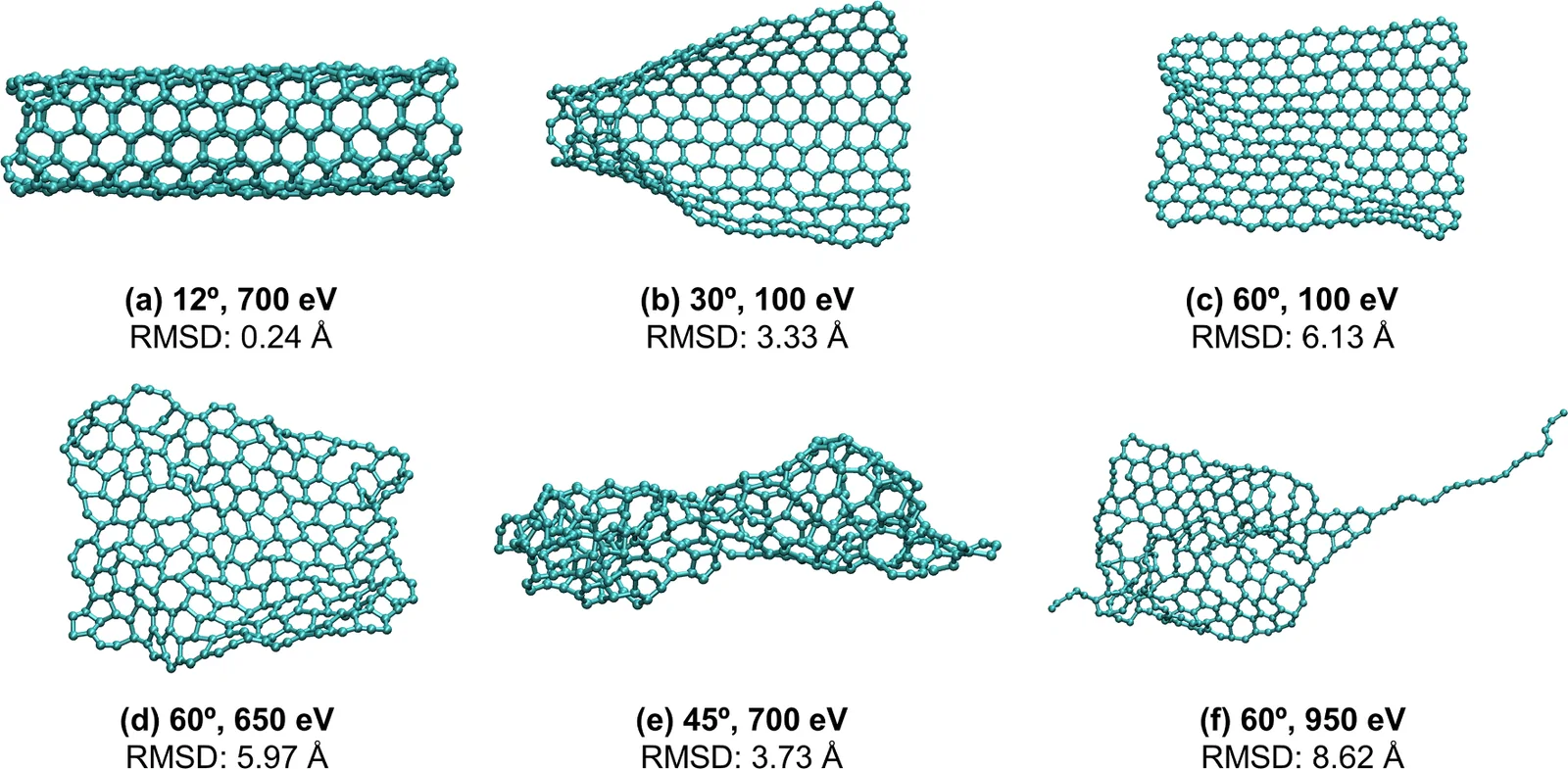
Representative structural outcomes obtained from collision-driven transformations towards the armchair edge of C$_{296}$ graphene nanoflakes, illustrating selected metastable morphologies with distinct topologies. The incidence angle θ and collision energy *E* for each trajectory are indicated. **a** Fully stitched nanotube exhibiting minimal deviation from the reference tubular geometry. **b** Partially closed tubular segment with incomplete seam propagation and one open end. **c** Non-planar nanoflake formed through bending without edge-to-edge bonding. **d** Non-planar flake with pronounced local defects and non-hexagonal ring motifs near the impact region. **e** Amorphous aggregate characterised by dense three-dimensional packing and loss of extended sheet or tubular topology. **f** Amorphous structure featuring extended, undercoordinated linear carbon tails protruding from a disordered core. RMSD values are reported relative to the corresponding reference nanotube geometry

The ensemble of collision trajectories reveals a rich landscape of structural outcomes. The finite GN evolves into a range of metastable structures that arise from the interplay between local bond reorganisation, global elastic deformation, and irreversible energy dissipation. Representative examples of these regimes are shown in Fig. 2, which highlights the principal topological classes observed under collision conditions that do not preferentially promote coherent tubular closure. Although all structures in the figure are drawn from impacts towards the armchair edge, qualitatively similar morphologies are also observed for zigzag edge-aimed collisions.

The first class (Fig. 2a) comprises fully stitched nanotubes, which yield the smallest RMSD relative to the reference tubular geometry across the entire dataset. In this regime, edge-to-edge contact is established across the full width of the nanoflake, and successive bond formation propagates away from the impact region to produce a continuous, low-defect seam and a nearly uniform cylindrical cross section. These structures exhibit the highest structural fidelity to the reference nanotube geometry across all simulated trajectories and serve as reference cases for successful collision-driven transformation from a nanoflake to a nanotube.

The second class (Fig. 2b) comprises partially closed tubes. Here, local stitching is initiated near the collision site, but seam propagation terminates before spanning the full length. The resulting morphologies feature one closed or partially capped end, where incomplete bonding reduces but does not eliminate the opening, and one open edge, producing tubular segments that retain strong curvature but are not fully closed. These configurations indicate that the initial onset of bending and local bond rearrangement can proceed without guaranteeing global closure, highlighting the need for deformation to propagate across the flake to bring distant edges into sustained contact and support continued seam growth.

The third class (Fig. 2c) consists of non-planar nanoflakes. In these structures, the dominant response is bending of the entire nanoflake rather than local bond rearrangement near the impact site. The flake adopts saddle-like or S-shaped conformations, with significant out-of-plane distortion but without the formation of persistent edge-to-edge bonds. These structures retain an open topology and reflect the relaxation of the deposited energy into low-frequency flexural modes (out-of-plane bending of the nanoflake), rather than into pathways leading to structural closure.

The fourth class (Fig. 2d) includes non-planar flakes with pronounced local defects. In this regime, bond rearrangements are concentrated near the impact region, leading to the formation of non-hexagonal rings and localised structural reconstruction, while the remainder of the flake undergoes out-of-plane bending similar to that observed in Fig. 2c. The resulting structures combine extended curvature with defect-rich patches, marking an intermediate state between reversible bending and irreversible topological transformation.

The fifth class (Fig. 2e) corresponds to compact amorphous aggregates, which are characterised by dense, three-dimensional packing of carbon atoms, irregular coordination environments, and the loss of an identifiable sheet or tubular backbone. Although short-range connectivity is preserved, long-range topological order is largely absent, indicating the formation of an amorphous carbon structure.

Finally, the sixth class (Fig. 2f) comprises amorphous morphologies featuring extended, undercoordinated “linear” tails. In these configurations, compact disordered cores coexist with chain-like carbon fragments that grow from the main cluster. These tails arise from incomplete bond saturation during rapid reorganisation and represent a distinct topological signature of non-equilibrium, defect-dominated pathways.

These six classes define a classification of structurally distinct outcomes that captures the dominant responses of the nanoflake to ballistic collisions with C$_{60}$. Rather than a simple dichotomy between open and closed structures, the system exhibits a hierarchy of intermediate morphologies that arise under different kinematic conditions, including structural bending, seam formation, defect generation, and amorphisation.

**Fig. 3:**
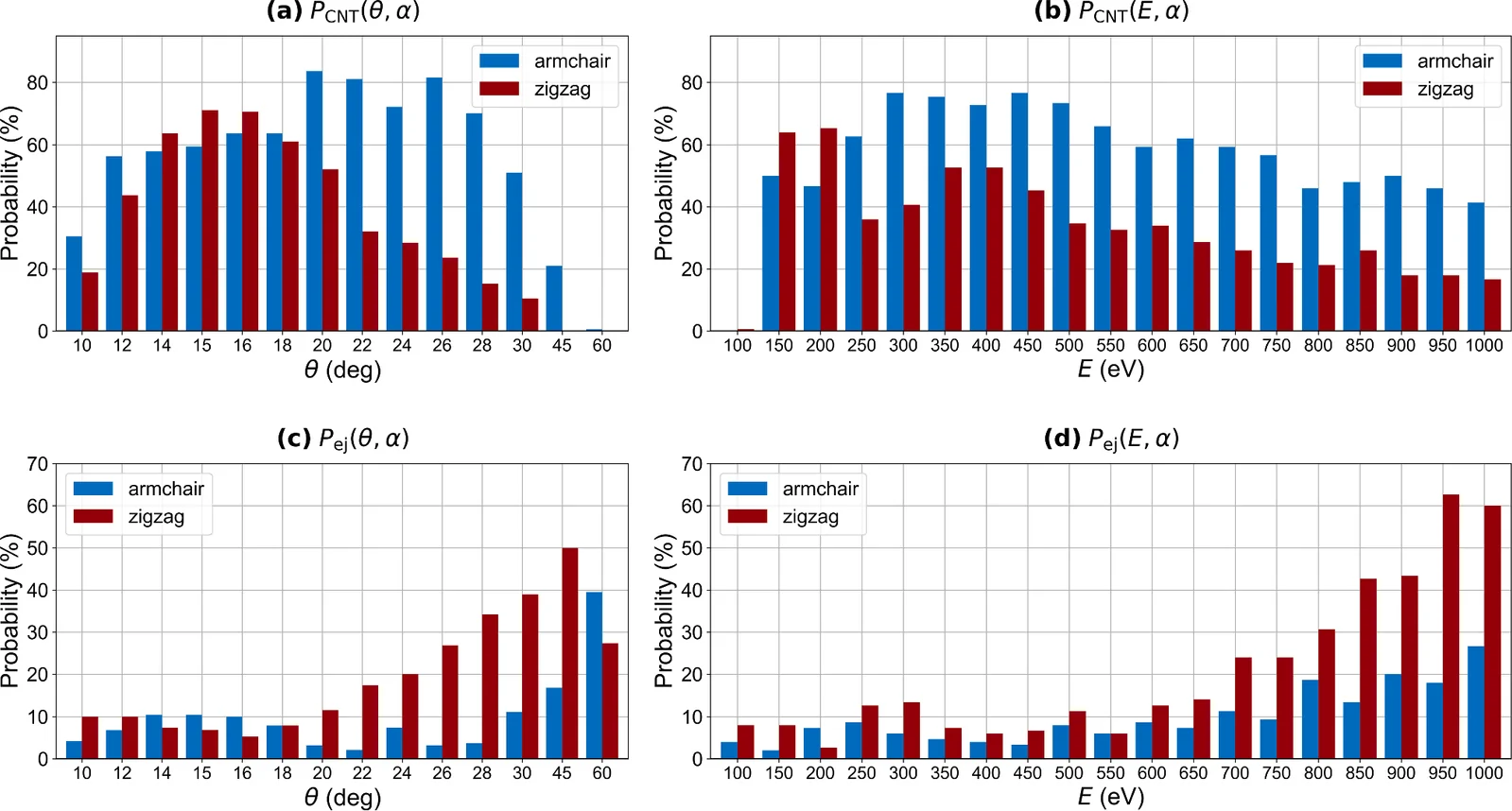
Statistical characterisation of collision outcomes for C$_{60}$ impacts on armchair and zigzag edges of C$_{296}$ graphene nanoflakes. Panels **a** and **b** report the angle- and energy-resolved probabilities of nanotube-like formation, $P_{\textrm{CNT}}(\theta ,\alpha )$ and $P_{\textrm{CNT}}(E,\alpha )$. Panels **c** and **d** show the corresponding probabilities of atom-ejection events, $P_{\textrm{ej}}(\theta ,\alpha )$ and $P_{\textrm{ej}}(E,\alpha )$. Blue and red bars denote armchair and zigzag edge-oriented collisions, respectively. The four panels highlight the distinct response of the two edge topologies, including the broader angular and energetic window for nanotube-like formation in armchair-directed collisions and the higher probability for atom ejection in the zigzag counterpart

This qualitative landscape is supported by the statistical trends summarised in Fig. 3. Panels (a) and (b) report the angle- and energy-resolved probabilities of nanotube-like formation, $P_{\textrm{CNT}}(\theta ,\alpha )$ and $P_{\textrm{CNT}}(E,\alpha )$. These probabilities correspond to the fraction of trajectories that yield nanotube-like structures, defined by an RMSD lower than 2 Å with respect to the reference nanotube geometry, following averaging over all sampled collision energies or incidence angles, respectively.

Across the full set of simulations spanning collision energies and incidence angles, the probability of nanotube-like formation is consistently higher for trajectories in which the projectile interacts primarily with the armchair edge than for those oriented towards the zigzag edge. Impacts directed at the armchair edge induce bending that brings the opposing zigzag edges into close proximity, facilitating their subsequent edge-to-edge stitching and coherent seam formation. Conversely, collisions towards the zigzag edge bring opposing armchair edges into proximity, where closure is less efficient. This trend is reflected in the angle- and energy-resolved probabilities shown in Fig. 3, where armchair-directed collisions exhibit a broader and more favourable window for nanotube-like transformation. The underlying origin can be rationalised in terms of the geometric compatibility of opposing zigzag edges, whose quasi-linear arrangement of undercoordinated carbon atoms favours sequential bond formation and coherent seam propagation once brought into alignment. By contrast, the corresponding alignment of armchair edges is less conducive to sustained stitching, leading to lower nanotube formation probabilities. In addition, zigzag graphene edges are known to exhibit localised electronic states near the Fermi level that confer partial radical character on the edge carbon atoms [74, 75], which is consistent with their higher reactivity and the enhanced probability for bond formation observed here.

Quantitatively, zigzag edge collisions display a well-defined maximum at $\theta = 15^\circ$, where $P_{\textrm{CNT}}$ reaches 71%, followed by a systematic decrease with increasing incidence angle, falling to 32% at $\theta = 22^\circ$ and 11% at $\theta = 30^\circ$, and vanishing at larger angles. Armchair-edge collisions exhibit a similar trend up to $\theta = 18^\circ$, where $P_{\textrm{CNT}}$ attains 64%, but subsequently develop a pronounced maximum, with values in the 80–85% range for $\theta = 20^\circ$–$26^\circ$. The energy-resolved statistics in Fig. 3b reveal a similarly broad window of high transformation probabilities for armchair-directed impacts, with $P_{\textrm{CNT}}$ remaining within 70–77% over the interval E=300–500 eV, followed by a gradual decline at higher energies. Zigzag edge collisions show a more heterogeneous response, with comparatively high probabilities at low energies, reaching 60–65% for E=150–200 eV, but decreasing steadily with increasing energy, dropping below 20% at the highest energies considered.

Complementary insight is provided by panels c and d of Fig. 3, which report the probabilities of atom-ejection events as functions of incidence angle and collision energy. Zigzag-directed collisions exhibit systematically higher ejection probabilities than armchair-directed collisions across a broad range of conditions. As a function of incidence angle (Fig. 3c), zigzag-directed collisions show a marked increase beyond $\theta = 20^\circ$, rising from 12% at $\theta = 20^\circ$ to 39% at $\theta = 30^\circ$ and reaching 50% at $\theta = 45^\circ$, whereas armchair edges remain below 10% over most of this range, only increasing significantly at larger angles (e.g. 40% at $\theta = 60^\circ$). A similar contrast is observed as a function of collision energy (Fig. 3d), where zigzag edges display a steady increase in ejection probability at higher energies, reaching values above 40% for E≥850 eV and peaking near 60% at E=1000 eV, while armchair edges exhibit a more moderate increase, remaining below 20% up to E=900 eV and rising to 27% at the highest energy. This asymmetry indicates a greater probability for bond rupture and structural degradation at zigzag terminations, which directly competes with and suppresses the formation of nanotube-like structures observed in panels a and b. Consistent with their higher reactivity [74, 75], collisions directed towards zigzag edges more readily induce bond breakage and atom ejection than those impacting armchair edges, thereby hindering the cooperative bond formation required for sustained seam propagation.

The structural motifs in Fig. 2 and the statistical trends in Fig. 3 establish a coherent picture in which topological closure arises from a competition between deformation-driven self-organisation and irreversible energy dissipation through bond rupture, defect formation, and atom ejection. This balance is strongly modulated by edge topology and impact geometry, which together determine whether the system evolves towards fully stitched nanotube-like structures, partially closed tubular segments, globally bent sheets, or disordered carbon networks.

### Statistical maps of collision-driven transformations

**Fig. 4:**
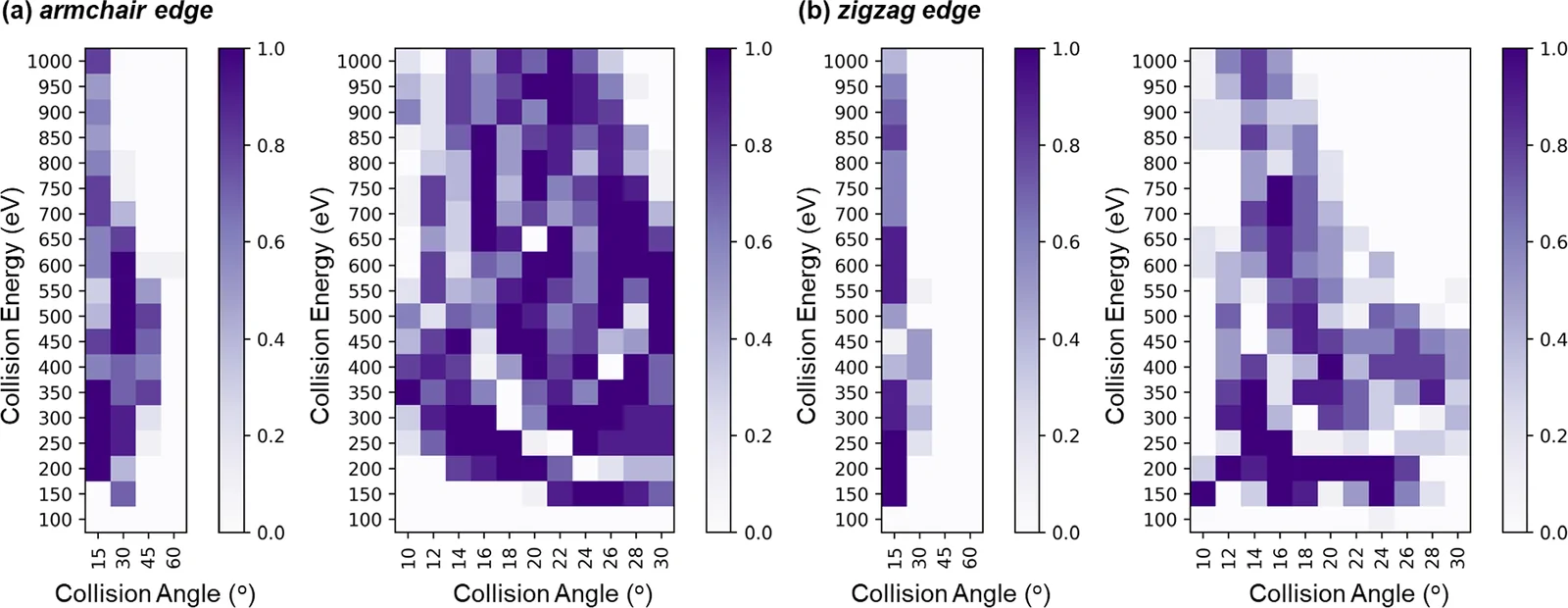
Probability maps for successful transformation to nanotube-like structures, $P_{\textrm{CNT}}$, as a function of collision energy and incidence angle for (**a**) armchair and (**b**) zigzag edge orientations of the C$_{296}$ graphene nanoflake. For each edge type, the left panel shows a coarse angular overview at representative angles of 15$^\circ$, 30$^\circ$, 45$^\circ$, and 60$^\circ$, while the right panel provides a high-resolution view of the low-angle regime (10$^\circ$–30$^\circ$) sampled at 2$^\circ$ increments. Each bin corresponds to a single (E,θ) parameter set and is colour-coded according to the bar scale, representing the probability of satisfying the criterion for nanotube-like structure formation, ranging from 0 (white) to 1 (dark purple). Regions of high transformation probability ($P_{\textrm{CNT}} \gtrsim 0.6$) delineate intermediate-energy and low-to-moderate-angle windows—typically around E≈200–$700~\textrm{eV}$ and $\theta \approx$ 10$^\circ$–30$^\circ$ for armchair-edge impacts—in which cooperative bending, edge-to-edge contact, and subsequent seam formation are most likely to occur

Figure 4 summarises the probability of successful transformation to nanotube-like structures, $P_{\textrm{CNT}}(E,\theta ,\alpha )$, across the explored collision parameter space. In contrast to Fig. 3a, b, which present angle- and energy-resolved probabilities obtained by averaging over *E* and θ, respectively, these maps retain the full (E,θ) resolution, thereby revealing the detailed distribution of high- and low-probability regions. The maps are organised by edge orientation, with panel a showing collisions to the armchair edge and panel b, to the zigzag edge. For each edge type, the left map provides a coarse angular overview at representative incidence angles (15$^\circ$, 30$^\circ$, 45$^\circ$, and 60$^\circ$), while the right map resolves the low-angle regime (10$^\circ$–30$^\circ$) at 2$^\circ$ increments. Each pixel corresponds to a single (E,θ) bin and is colour-coded according to $P_{\textrm{CNT}}$ from 0 (white) to 1 (dark purple), where 1 indicates that all trajectories within the corresponding bin result in nanotube-like structures satisfying the RMSD-based success criterion.

Across both edge orientations, the maps display a pronounced non-monotonic dependence on collision energy. At the lowest energy considered (100 eV), $P_{\textrm{CNT}}$ is essentially zero across the entire angular range, indicating that the momentum transferred to the nanoflake during the collisions is insufficient to trigger the sequence of structural rearrangements required for nanotube closure. Already at 150 eV, however, pronounced high-probability domains emerge in collisions aimed at both armchair and zigzag edges. In the two-dimensional maps, these localised regions reach $P_{\textrm{CNT}} \approx 0.8$–1 for selected collision angles, spanning $\theta \approx 22$–30$^\circ$ for the armchair edge and occurring more sparsely for the zigzag edge (notably at $\theta \approx 10^\circ$, 15–18$^\circ$, and ∼24$^\circ$). Consistently, the angle-averaged probabilities increase to 0.50 for armchair and 0.64 for zigzag edge orientations.

Upon further increasing the collision energy, the armchair case develops a broad favourable window extending approximately from 250 to 750 eV, within which several angular regions exhibit $P_{\textrm{CNT}} > 0.8$, and the averaged probability remains in the range 0.60–0.77. In contrast, the zigzag case shows a narrower and more fragmented pattern, with its largest probabilities concentrated at 150–200 eV and within a more restricted angular range of $\theta \approx 10^\circ$–26$^\circ$.

At higher collision energies (E≥750 eV), the probability of nanotube-like formation decreases again, as evidenced by the re-emergence of low-probability regions in the maps and by the reduction of the averaged $P_{\textrm{CNT}}$ to about 0.41–0.50 for armchair and 0.17–0.26 for zigzag at 800–1000 eV. This trend reflects the increasing contribution of competing processes such as extensive bond rupture, atom ejection, and the formation of highly defective configurations that do not satisfy the criterion for nanotube-like structures. The region of largest $P_{\textrm{CNT}}$ therefore lies at intermediate energies, but its angular extent and the continuity of high-probability regions depend strongly on edge orientation.

For the armchair edge (Fig. 4a), the coarse-angle overview indicates that successful transformation is concentrated at the smaller representative angles (15$^\circ$–30$^\circ$), whereas large incidence angles strongly suppress closure. In particular, the 60$^\circ$ column exhibits near-zero probabilities across the full energy range, with only a single isolated bin (600 eV) reaching $P_{\textrm{CNT}} = 0.1$. Likewise, the 45$^\circ$ column is predominantly characterised by low values ($P_{\textrm{CNT}} \le 0.1$), except in the 350–550 eV range, where $P_{\textrm{CNT}}$ typically increases to 0.4–0.7. By contrast, the 15$^\circ$ and 30$^\circ$ columns display extended energy intervals with consistently high transformation probabilities. For these angles, $P_{\textrm{CNT}}$ frequently exceeds 0.7 and reaches values close to unity over contiguous energy ranges, indicating that such incidence angles provide favourable conditions for cooperative bending and subsequent edge-to-edge stitching.

The high-resolution map for the armchair case (Fig. 4a, right) reveals that the low-angle regime is not uniformly favourable but instead exhibits a structured distribution of high- and low-probability regions. Several extended patches of moderate-to-high $P_{\textrm{CNT}}$ ($0.5 \le P_{\textrm{CNT}} \le 1.0$) appear within 10$^\circ$–30$^\circ$, separated by lighter “corridors” where the probability decreases markedly. The presence of these “gaps” indicates that, even within this nominally favourable angular range, small variations in θ can redirect the collision outcome towards competing channels that deviate from the reference nanotube topology. Part of this variability may also arise from the limited statistical sampling within individual (E,θ) bins. Notably, elevated $P_{\textrm{CNT}}$ values are not confined to a narrow angular interval but extend across a substantial fraction of the 10$^\circ$–30$^\circ$ range, indicating that collisions directed at the armchair edge support successful nanotube closure over a broad set of incidence angles once the energy is sufficient to induce bending and initiate edge-to-edge stitching.

For the zigzag edge (Fig. 4b), the overall probability is confined to more selective regions of the parameter space. The coarse-angle overview already indicates a stronger sensitivity to the incidence angle θ: the probabilities at 60$^\circ$ and 45$^\circ$ remain effectively zero across the entire energy range. At 15$^\circ$, however, the zigzag edge remains competitive with the armchair case, with extended energy intervals displaying moderate-to-high $P_{\textrm{CNT}}$ values. This favourable behaviour is not maintained at 30$^\circ$, for which the probability decreases much more strongly and high-$P_{\textrm{CNT}}$ values become markedly less frequent than in the armchair case.

This selectivity becomes clearer in the high-resolution zigzag map (Fig. 4b, right), which exhibits distinct, well-defined bands rather than a broad plateau. A prominent feature is a high-probability band at low energies (approximately E≈150–200 eV), extending across small incidence angles (about $\theta \approx 10^\circ$–26$^\circ$), indicating a regime where a modest collision-induced momentum transfer to the target can already promote nanotube closure when the geometry is favourable. At higher energies, the map resolves into inverted diagonal bands, in which the high-probability regions shift towards lower incidence angles as the collision energy increases. In particular, probabilities remain appreciable around $\theta \approx 26^\circ$–30$^\circ$ for E≈350–450 eV, but at higher energies (E≈500–700 eV) they become largely confined to $\theta \approx 12^\circ$–20$^\circ$.

A defining feature of the zigzag case is the pronounced depletion of $P_{\textrm{CNT}}$ in the upper-right segment of the map (large θ, high *E*), where extended low-probability regions dominate. This contrasts with the armchair map, which retains substantial probabilities in the same region, and is consistent with the sharper decline observed in the angle-averaged trends (Fig. 3b). Overall, the zigzag map exhibits a fragmented distribution, in which favourable conditions for nanotube closure are restricted to narrower and more weakly connected regions of the parameter space.

Direct comparison of panels a and b shows that armchair edge-oriented collisions provide a wider low-angle parameter space over which high $P_{\textrm{CNT}}$ can be achieved, whereas collisions towards the zigzag edge confine successful nanotube closure to narrower windows and more weakly connected regions. This contrast is particularly evident at higher energies, where the armchair configuration continues to support appreciable $P_{\textrm{CNT}}$ across a broad angular range, while the zigzag case is largely suppressed outside a limited set of low-angle conditions.

This trend is consistent with the edge-dependent mechanisms discussed above, namely the favourable geometric alignment that promotes efficient stitching of opposing zigzag edges following armchair-directed impacts. The efficiency of seam formation therefore depends not only on the availability of reactive, unpassivated edge atoms but also on the local topology of the edge segments, which determines the formation of bridging bonds during the final stitching stage. In practical terms, the probability maps indicate that successful nanotube formation is determined by a balance between (i) sufficient momentum transfer to induce bending and bring the opposing edges into contact, and (ii) avoidance of excessive local energy deposition and tangential shear of the nanoflake, which promote competing processes and suppress coherent seam propagation. The maps thus provide a quantitative partition of the explored parameter space into regimes of suppressed transformation, partial transformation, and high-probability nanotube-like structure formation, which are used in the following section to select representative trajectories for mechanistic analysis.

**Fig. 5:**
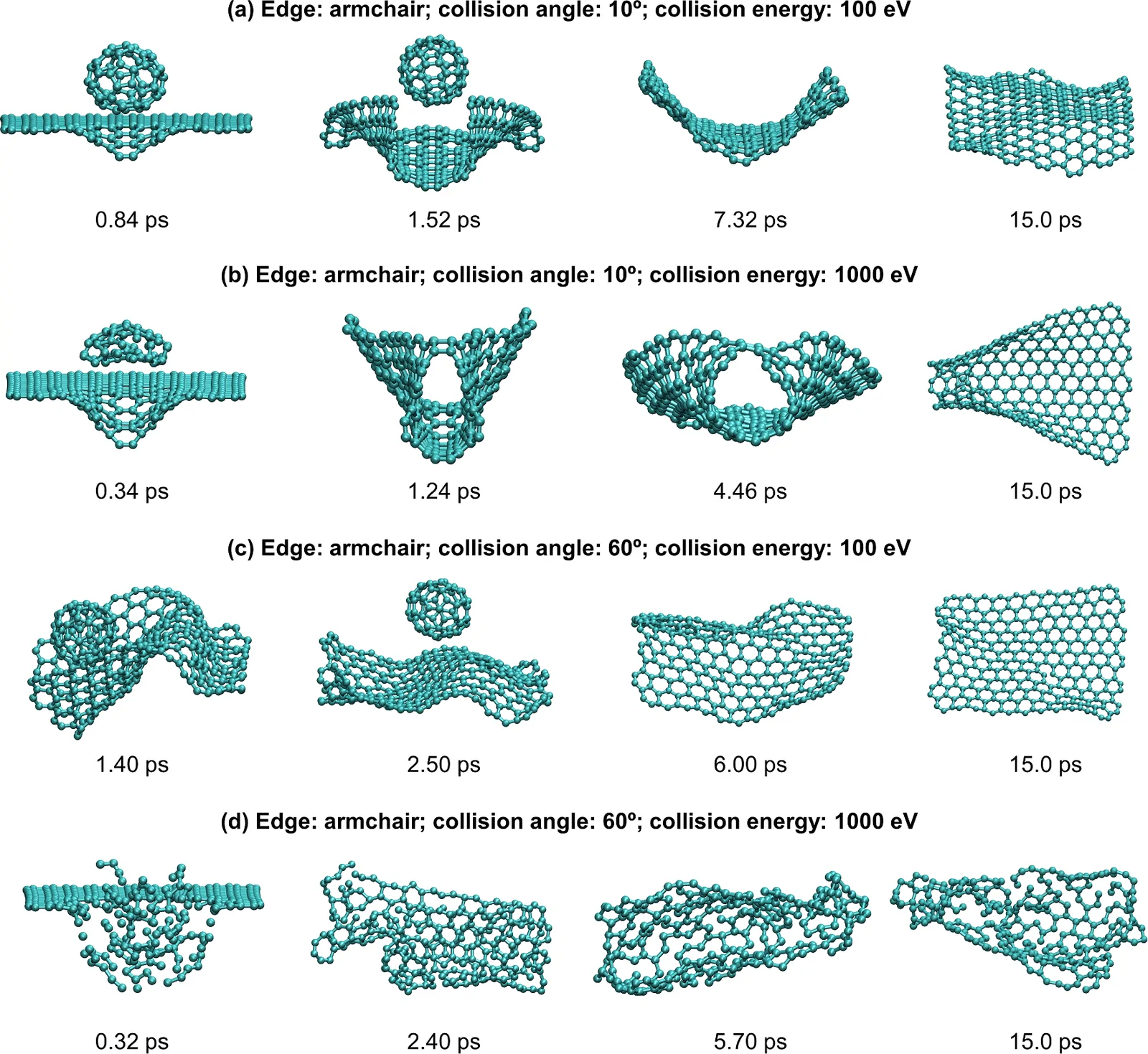
Representative armchair edge-aimed collision-induced outcomes in regimes that do not lead to the formation of nanotube-like structures. **a** Low incidence angle (10$^\circ$) and low collision energy (100 eV): the fullerene traverses the flake, inducing transient bending and global non-planar distortion without driving the opposing edges into contact, yielding an open, deformed nanoflake. **b** Low incidence angle (10$^\circ$) and high collision energy (1000 eV): excessive local energy deposition produces extensive bond reorganisation and the formation of a four-membered ring bridging the two sides of the flake, together with a large, irregular aperture, resulting in an asymmetric, defect-rich cluster with a partially capped and an open end. **c** High incidence angle (60$^\circ$) and low collision energy (100 eV): the momentum is transferred into long-wavelength flexural modes, generating an S-shaped deformation and rebound of the projectile without edge-to-edge bonding. **d** High incidence angle (60$^\circ$) and high collision energy (1000 eV): severe and irreversible disruption of both projectile and target leads to atom ejection (36 atoms removed from the nanoflake, yielding C$_{260}$) and the formation of a highly defective, loosely tubular cluster. Snapshots are shown at selected instants of each simulated trajectory, illustrating the progression from initial impact to the final metastable or defective morphology at 15 ps

To elucidate the physical origin of the low-probability regions in the probability maps, Fig. 5 presents representative trajectories for collisions to the armchair edge sampled from four distinct regions of the (E,θ) space that do not lead to the formation of a nanotube-like structure. These cases illustrate how different partitions of energy and momentum at impact redirect the system towards non-closure pathways, thereby shaping the low- and high-probability domains observed in Fig. 4.

At $\theta = 10^\circ$ and E=100 eV (see Fig. 5a), the fullerene traverses the nanoflake with the smallest momentum transfer normal to the nanoflake plane, as the component of the impact momentum perpendicular to the plane increases with the incidence angle ($p_{\perp } = p \sin \theta$). This modest transfer is consistent with the limited bending induced and the low probability of nanotube-like closure observed under these conditions. The early snapshots (at ∼0.8 and 1.5 ps) show transient local bending and shallow indentation beneath the projectile, followed by lateral strain propagation that produces a pronounced, saddle-like distortion of the flake. However, the transferred momentum is insufficient to bring the opposing edges into proximity. As a result, the nanoflake relaxes into a non-planar, topologically open configuration. This regime corresponds to the pale, low-$P_{\textrm{CNT}}$ bins at the bottom of the energy axis in Fig. 4a, where elastic deformation and reversible structural rearrangements at the edges dominate over cooperative seam formation.

For $\theta = 10^\circ$ and E=1000 eV (Fig. 5b), the collision results in substantial and highly localised energy deposition at the impact site. The fullerene penetrates the nanoflake and rebounds, generating extensive bond rupture and rapid local network reorganisation in the impact zone. The snapshots reveal the formation of a four-membered ring that bridges the two sides of the flake, accompanied by the opening of a large, irregular aperture in the local network. While one side of the nanoflake undergoes partial closure into a capped, defective tubular segment, the excess energy is not effectively transferred to the opposite side, which remains open, yielding an asymmetric structure with localised edge-to-edge bonding but no continuous seam. This outcome exemplifies a competing channel at high collision energies, where excessive local energy deposition favours misaligned bond formation and defect accumulation rather than coherent, edge-to-edge seam propagation. In the probability maps shown in Fig. 4, this behaviour contributes to the re-emergence of low-$P_{\textrm{CNT}}$ regions at the upper part of the energy axis.

At $\theta = 60^\circ$ and E=100 eV (Fig. 5c), the collision occurs at a steep incidence angle, providing a substantial momentum component normal to the nanoflake plane. However, the low kinetic energy of the projectile is insufficient to induce sustained curvature or to drive the opposing edges into contact. Instead, the fullerene induces a pronounced S-shaped flexural response of the nanoflake, with the deposited energy redistributed into low-frequency bending modes involving out-of-plane deformation of the entire nanoflake, rather than being localised near the edges. The projectile subsequently drifts and rebounds, but the opposing sides of the flake never approach closely enough to initiate bonding. The final structure remains an open, non-planar fragment, consistent with the extensive low-$P_{\textrm{CNT}}$ regions observed in the 45$^\circ$ and 60$^\circ$ columns of Fig. 4a. This regime highlights the role of incidence angle in funnelling energy into global vibrational motion that relieves strain without promoting nanotube closure.

Finally, for $\theta = 60^\circ$ and E=1000 eV (Fig. 5d), the combination of steep incidence and high kinetic energy of the projectile leads to extensive, irreversible fragmentation of both the projectile and the target. The fullerene is destroyed upon impact, and a substantial fraction of the nanoflake undergoes bond rupture and atom ejection. In the representative trajectory shown in Fig. 5d, 36 atoms are emitted from the nanoflake, yielding a highly defective C$_{260}$ fragment. Although this structure exhibits a loosely tubular envelope, it is characterised by a high density of vacancies, non-hexagonal motifs, and disrupted connectivity, and remains in this defective state throughout the simulation. This regime corresponds to the extreme high-energy limit of the maps, where fragmentation and amorphisation dominate over cooperative seam formation, resulting in near-zero $P_{\textrm{CNT}}$ across all large incidence angles.

These four regimes provide a mechanistic framework for understanding collision-induced transformations in finite-size carbon nanosystems. At low collision energies, elastic and flexural responses dominate, dissipating the transferred momentum into global deformation without enabling edge-to-edge contact. At high collision energies, the dynamics increasingly favour extensive bond rupture, defect formation, and atom ejection, as excessive local energy deposition disrupts coherent seam propagation. Successful transformation into a nanotube-like structure is therefore confined to an intermediate regime, in which the normal component of the transferred momentum is sufficient to induce cooperative bending and bring opposing edges into proximity, while remaining moderate enough to preserve the structural integrity required for sustained stitching. The dependence on edge orientation reflects the interplay between geometry and reactivity. Armchair-directed impacts align opposing zigzag edges, facilitating efficient bridging and seam propagation, whereas zigzag-directed impacts bring opposing armchair edges into contact, a configuration that promotes less coherent closure and is more prone to disruption.

### Mechanism of edge-mediated topological closure

**Fig. 6:**
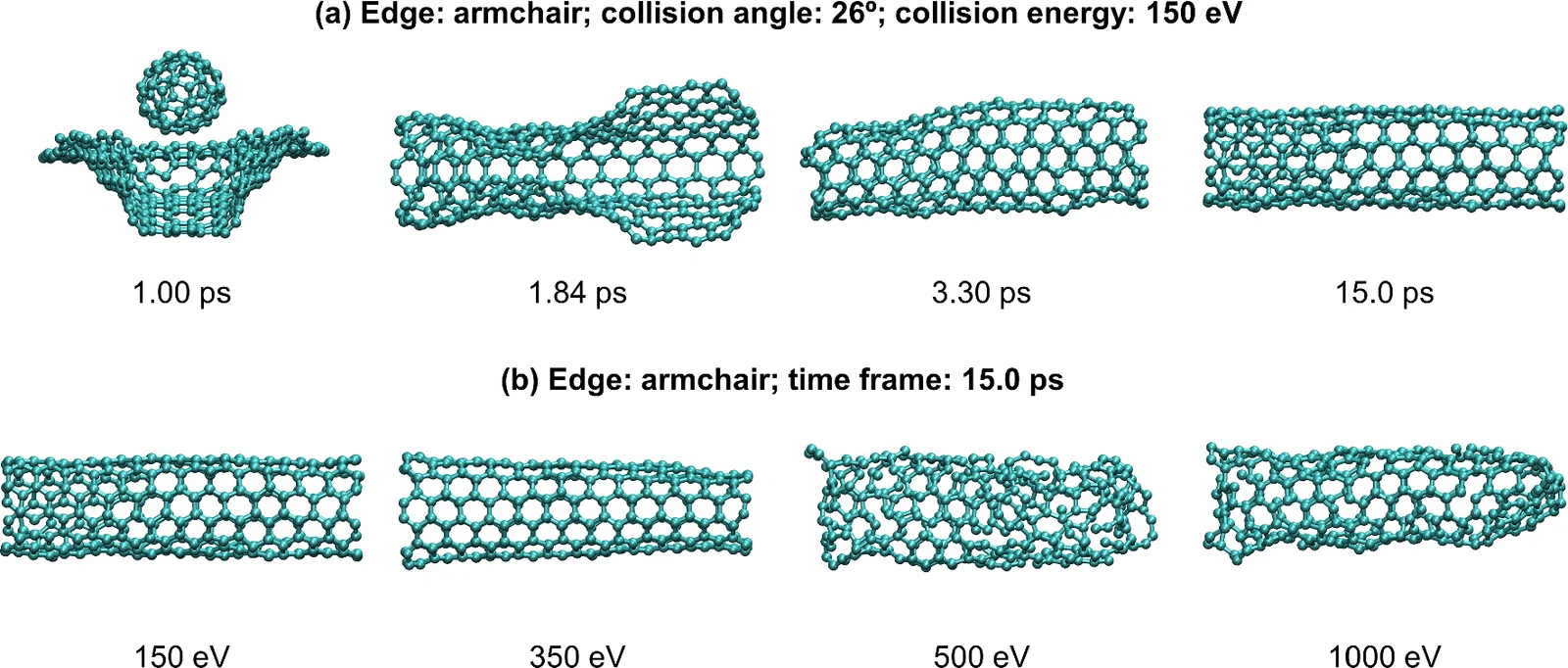
Mechanism of edge-mediated topological closure of a graphene nanoflake from a fullerene collision aimed at the armchair edge of a C$_{296}$ graphene nanoflake following ballistic impact. **a** Time-resolved snapshots for a representative trajectory at an incidence angle of 26$^\circ$ and a collision energy of 150 eV, illustrating the sequence from initial edge contact and grazing penetration of the C$_{60}$ projectile, through local bending and progressive, zipper-like seam formation between opposing zigzag edges, to the emergence of a fully connected nanotube by t=15.0 ps. The simulation snapshot at intermediate instant (t≈1.84 ps) highlights the systematic propagation of edge-to-edge bonding away from the impact region. **b** Final morphologies obtained at increasing collision energies (150, 350, 500, and 1000 eV) for the same edge orientation, showing the transformation from open-ended nanotubes at lower energies to closed, cage-terminated nanotubes at higher energies, driven by enhanced post-closure energy redistribution and secondary bond reorganisation at the tube extremities

Figure 6 illustrates the atomistic sequence by which a finite, hydrogen-free graphene nanoflake undergoes coherent topological closure following a ballistic impact, using a representative armchair-oriented case at an incidence angle of 26$^\circ$ and a collision energy of 150 eV. The time-resolved snapshots in Fig. 6a reveal a strongly localised, yet system-spanning, mechanism in which energy deposited at a single edge site is redistributed across the entire flake, ultimately driving a coordinated, edge-to-edge stitching process that converts a planar fragment into a fully connected nanotube.

At early simulation times (t≈1.00 ps), the C$_{60}$ projectile makes initial contact with the nanoflake near the armchair edge and slides along the flake surface while partially penetrating the edge region. This grazing interaction provides sufficient normal momentum transfer at the nanoflake edge while avoiding the excessive local energy density associated with fragmentation. The immediate response is the formation of a shallow curvature and local rehybridisation at the impact site, accompanied by the nucleation of non-hexagonal motifs and transient $sp^2$–$sp^3$-like bonding that act as seeds for structural bending.

By t≈1.84 ps, the deformation front has propagated away from the collision zone, and the opposing zigzag edges are drawn into close proximity. Rather than closing a nanotube at a single point, the snapshots show a progressive, zipper-like seam formation that initiates near the impact region and advances systematically along the length of the nanoflake. This stage reflects a cooperative process in which local bond formation at the edge reduces the number of unsaturated carbon sites, providing a strong energetic driving force for continued stitching. The result is a rapid transition from a bent, open fragment to a contiguous, tubular topology.

At intermediate times (t≈3.30 ps), the seam has propagated across the full width of the flake, yielding a fully stitched nanotube-like structure. The residual kinetic energy is redistributed into global vibrational modes of the newly formed nanotube, leading to minor radial breathing and axial oscillations rather than further topological rearrangement. By the end of the simulated trajectory (t=15.0 ps), the system has relaxed into a perfectly connected nanotube with no persistent projectile attachment and minimal residual defects, satisfying the nanotube formation criterion defined in Sec. 2.

Figure 6b extends this mechanistic picture across a broad energy range by comparing the final morphologies obtained at 150, 350, 500, and 1000 eV for armchair edge-oriented collisions at representative incidence angles ($\theta = 26^\circ$, 10$^\circ$, 45$^\circ$, and 22$^\circ$, respectively). At the two lower energies (150 and 350 eV), the resulting nanotubes retain open ends. In these cases, the energy transfer is sufficient to induce bending and drive seam propagation along the opposing edges, but the remaining kinetic energy after closure is not sufficient to promote further bond rearrangements at the tube termini. The open rims therefore persist as stable, metastable edge structures over the full simulation window.

In contrast, at higher energies (500 and 1000 eV), the nanotubes undergo additional structural evolution at their ends, leading to complete closure. In these cases, the more intense initial energy deposition not only drives rapid seam formation across the entire nanoflake but also leaves a significant amount of internal energy within the newly formed nanotube. This excess energy is subsequently redistributed towards the open ends, where it promotes further bond rearrangements, the formation of non-hexagonal rings, and eventual cap formation. The result is the emergence of closed, cage-terminated nanotubes, indicating a transformation from edge-mediated seam propagation to a two-stage process in which seam formation is followed by end capping driven by internal energy redistribution.

Beyond the structures directly observed here, the same collision-driven closure mechanism may provide routes to more complex carbon nanostructures. Stable C$_{60}$ encapsulation is unlikely for the present C$_{296}$ nanoflake because the resulting nanotube-like structures are too narrow to accommodate the fullerene without substantial distortion. However, larger nanoflakes forming wider nanotubes may enable collision-driven formation of C$_{60}$@CNT complexes. More generally, closure around smaller projectiles or projectile fragments, including metal nanoclusters, could produce endohedral tubular structures if encapsulation occurs before their escape from the curved nanoflake. An analogous mechanism involving the coordinated closure of bilayer graphene flakes could potentially generate double-walled nanotube-like structures. Such processes could provide access to metal-filled nanotubes, confined nanocluster–carbon hybrids, multiwalled structures, and other endohedral host–guest nanomaterials with potentially tuneable electronic, magnetic, and catalytic properties.

The simulated trajectories establish a clear picture of collision-induced topological transformation in graphene nanoflakes. At the atomic scale, local mechanical deformation at the impact region initiates bending of the nanoflake sheet. The target responds as an elastically deformable lattice that efficiently transmits the impact-induced strain across its span, bringing distant edge segments into contact. The newly formed nanotube redistributes the remaining energy into collective vibrational modes, which, depending on the initial collision energy, either stabilise open-ended tubes or promote further topological reorganisation towards fully closed morphologies.

This hierarchy of processes explains the energy- and angle-dependent probability landscapes reported in Fig. 4. For armchair-edge impacts, intermediate collision energies (approximately E≈200–$700~\textrm{eV}$) and low-to-moderate incidence angles (about $\theta \approx 10^\circ$–$30^\circ$) favour the cooperative regime in which elastic bending, edge contact, and subsequent seam propagation combine to enable coherent nanotube formation. At lower energies, particularly near $E \approx 100~\textrm{eV}$, the process typically stalls at the stage of curvature development without closure, whereas at higher energies (roughly $E \ge 800~\textrm{eV}$) it is increasingly overtaken by competing channels such as defect accumulation, atom ejection, and fragmentation. The mechanism identified here thus provides a unifying framework linking local edge chemistry, global mechanical response, and the emergent topology of collision-driven carbon nanostructures.

## Conclusion

In summary, we investigated collision-driven topological transformations in finite, hydrogen-free graphene nanoflakes to elucidate how strongly non-equilibrium energy input enables access to regions of the carbon allotrope landscape that remain inaccessible near equilibrium, including pathways associated with structural bending, bond rearrangement, and edge-to-edge stitching. Using reactive molecular dynamics simulations of ballistic impacts between a C$_{60}$ fullerene and a square C$_{296}$ nanoflake containing equal numbers of armchair and zigzag edge segments, we mapped the resulting collision outcome landscape and identified the conditions under which coherent nanotube-like formation becomes statistically favoured. The trajectory ensemble reveals a broad spectrum of metastable morphologies, including fully stitched nanotubes, partially stitched tubular segments, bent non-planar flakes, locally reconstructed defective structures, compact amorphous geometries, and highly disordered carbon networks featuring undercoordinated chain-like tails. This diversity reflects a competition between cooperative bending and irreversible energy dissipation through bond rupture, defect formation, and atom ejection, the balance of which depends sensitively on incidence angle, collision energy, and edge topology.

Statistical analysis shows that armchair-oriented impacts sustain coherent tubular transformation over a substantially broader parameter window than zigzag-oriented impacts, as quantified by RMSD-based similarity to a reference nanotube and by the occurrence of atom ejection. The probability maps indicate a pronounced non-monotonic dependence on collision energy, with successful nanotube-like closure confined to low-to-moderate oblique incidence angles ($\theta \approx 10^\circ$–30$^\circ$) and intermediate collision energies (E≈200–700 eV). In this regime, the transferred momentum is sufficient to induce bending and bring opposing edges into contact, while remaining moderate enough to preserve the structural integrity required for sustained stitching. At lower energies, deformation remains largely elastic and does not lead to edge-to-edge bonding, whereas at higher energies the increasing contribution of bond rupture, defect formation, and atom ejection suppresses coherent closure. Zigzag-oriented impacts display narrower and more fragmented regions of high $P_{\textrm{CNT}}$, together with a stronger reduction of $P_{\textrm{CNT}}$ at high energies and systematically higher atom ejection probabilities, consistent with an increased tendency towards local bond rupture and defect accumulation that interrupts seam propagation.

Time-resolved trajectory analysis reveals an atomistic sequence for coherent edge-mediated closure. A grazing impact at the nanoflake edge initiates local bonding rearrangement and bending, after which elastic strain redistribution across the nanoflake brings distant edge segments into proximity. Nanotube closure proceeds through a zipper-like stitching process that propagates away from the impact region, forming a continuous seam and a structurally coherent nanotube. After the tubular topology is established, the remaining internal energy redistributes into collective vibrational motion of the nascent nanotube, which stabilises open-ended morphologies at lower collision energies but can promote closure of the nanotube ends at higher energies.

In a broader context, these results establish a non-equilibrium framework linking unpassivated edge chemistry, collision-induced energy transfer, and structural rearrangement in finite graphene fragments. The findings support collision-driven self-organisation as aviable physical route to tubular and cage-like carbon nanostructures in environments where thermal activation is suppressed, including low-temperature astrochemical settings. Future work will systematically vary nanoflake size and shape, incorporate finite-temperature initial conditions to quantify the role of thermal fluctuations, defect annealing, and thermally assisted barrier crossing, and explore repeated-collision protocols and alternative projectiles to assess the robustness and generality of the edge-mediated closure pathways identified here.

## Data Availability

This manuscript has associated data in a data repository. [Authors’ comment: The data that support the findings of this study are available from the corresponding author upon reasonable request.].
